# No maternal or direct effects of ocean acidification on egg hatching in the Arctic copepod *Calanus glacialis*

**DOI:** 10.1371/journal.pone.0192496

**Published:** 2018-02-07

**Authors:** Peter Thor, Fanny Vermandele, Marie-Helene Carignan, Sarah Jacque, Piero Calosi

**Affiliations:** 1 Norwegian Polar Institute, Fram Centre, Tromsø, Norway; 2 Université du Québec à Rimouski, Département de Biologie Chimie et Géographie, Rimouski, Canada; Evergreen State College, UNITED STATES

## Abstract

Widespread ocean acidification (OA) is transforming the chemistry of the global ocean and the Arctic is recognised as the region where this transformation will occur at the fastest rate. Moreover, many Arctic species are considered less capable of tolerating OA due to their lower capacity for acid-base regulation. This inability may put severe restraints on many fundamental functions, such as growth and reproductive investments, which ultimately may result in reduced fitness. However, maternal effects may alleviate severe effects on the offspring rendering them more tolerant to OA. In a highly replicated experiment we studied maternal and direct effects of OA predicted for the Arctic shelf seas on egg hatching time and success in the keystone copepod species *Calanus glacialis*. We incubated females at present day conditions (pH_T_ 8.0) and year 2100 extreme conditions (pH_T_ 7.5) during oogenesis and subsequently reciprocally transplanted laid eggs between these two conditions. Statistical tests showed no effects of maternal or direct exposure to OA at this level. We hypothesise that *C*. *glacialis* may be physiologically adapted to egg production at low pH since oogenesis can also take place at conditions of potentially low haemolymph pH of the mother during hibernation in the deep.

## Introduction

Uptake of anthropogenic CO_2_ is changing the chemistry of the global ocean [1]. When entering the sea, CO_2_ reacts with water to form carbonic acid, and this ocean acidification (OA) has lowered the global ocean mean surface pH from 8.13 during the pre-industrial age to the present day 8.05. This trend is predicted to continue and current models estimate a further decrease of up to 0.4 pH units by the year 2100 [2–4]. In this respect, the Arctic is a region of specific concern. OA is currently progressing at faster rates in many Arctic regions and is expected to continue to do so [1, 5–7]. This is partly due to rising temperatures generating increasing volumes of sea ice melt water, which holds low H^+^ buffering capacity [8]. Moreover, while the Arctic Ocean contains only 1% of the global ocean volume, it receives 11% of the global riverine discharge. This discharge not only carries low H^+^ buffering capacity but also contributes significant loads of terrestrial carbon, which increases CO_2_ production by promoting heterotrophic microbial respiration [9]. Finally, inflow from the North Atlantic transports increasing amounts of anthropogenic CO_2_ to the Arctic Ocean [10]. Arctic organisms are therefore the first to face the effects of OA and will continue to experience stronger OA in the future [7].

The magnitude of these changes extends beyond anything experienced during most species’ evolutionary history [11], and while significant effects are predicted for many marine animals [12, 13], effects may be particularly conspicuous in the Arctic. Contrary to lower latitude eurythermal animals, true Polar species show low energetic costs for physiological maintenance at low temperatures [14, 15]. This is, however, at the expense of a lower capacity for cellular homeostasis and acid-base regulation [16]. The capacity to counter negative effects of OA is therefore thought to be particularly low in Arctic species. Moreover, while direct effects from the immediate environment will impose serious implications for sensitive organisms, effects carried through the generations may convey either alleviation or additional stress. Adaptation will enable increased tolerance to a changed environment [17], while maternal effects carried to the offspring may act in either direction. Maternal effects can precondition offspring to the environment experienced by mothers through cytoplasmic and mitochondrial factors packaged into the egg during oogenesis [18–21]. However, under severe maternal stress these factors may be lacking or differently conditioned resulting in poorly developing offspring. For instance, copepods mothers’ ingestion of low quality prey including harmful diatoms have shown detrimental effects on egg hatching and subsequent naupliar development [22–24]. Such changes may have far-reaching consequences, and a depression of the long term year-to-year recruitment of nauplii larvae or a delay in the timing of this recruitment can have significant effects on the population development [25]. When these effects occur in ecologically important species, they may spread beyond the species itself.

*Calanus glacialis* constitutes a keystone species in the Arctic Ocean and adjacent seas [26–28]. Along the continental shelf this species dominates in terms of biomass, may exert significant grazing pressure on microplankton prey, and is a very important food item for many pelagic predators such as carnivorous copepods and amphipods, but also Arctic fish species, baleen whales, and marine birds [29–31]. Any changes to *C*. *glacialis* production will therefore extend beyond the copepods themselves and potentially encompass a larger part of the entire food web. As a consequence, much attention has been given *C*. *glacialis* and its possible future in a changing Arctic [32–36].

In the present study, we investigated the effects of the immediate and maternal pH environment as predicted for the Arctic Ocean in year 2100 [1] on egg hatching dynamics in *C*. *glacialis*. Egg hatching success (*EHS*) is not the only determinant, besides egg production rate, of population recruitment success. Copepod eggs are often subjected to high mortality rates [37–39], and while decreased *EHS* would have direct consequences for population recruitment, any delay in the timing of egg hatching will increase individual predation risk. Moreover, delayed hatching may increase the risk of phenological miss-match with the succession of the phytoplankton bloom rendering the developing larvae less adequately nourished [25]. We therefore investigated the effects of OA on both *EHS* and egg hatching time. Differentiation between maternal and direct effects was accomplished by reciprocally transplanting eggs produced by females subjected to high and low pH. *Calanus glacialis* show both capital and income breeding strategies [40, 41], so the study period was chosen late in spring to avoid contribution from the winter energy capital and increase the possibility that all energy for egg production was obtained from ingested food during the OA incubation.

## Methods

### Collection of copepods

*Calanus glacialis* were caught by vertical tows, 100 m to the surface, with a 200 μm WP2 plankton net (KC Research equipment, Silkeborg, Denmark) with a closed cod end in the Kongsfjord, Svalbard (79.0° N, 11.7° E) in late May 2016. No specific authorisation was needed for collecting copepods, and no endangered species were involved. On deck, the content of the cod end was diluted in 25 L sea water produced from water collected at 80 m. Copepods were then transported to a cold room (5°C) at the nearby Kings Bay Marine Laboratory (Ny-Ålesund, Svalbard). *Calanus glacialis* females were selected under the stereoscope using cut off plastic Pasteur pipettes keeping all vessels on ice to avoid high temperatures. Individuals were identified to species by number of pleopods and abdominal segments. They were distinguished from *C*. *finmarchicus* and *C*. *hyperboreus* on the basis of size [28], the presence of red pigmentation in the antennules, a characteristic distinguishing *C*. *glacialis* from *C*. *finmarchicus* [42], and the lack of lateral spikes on the distal prosome segment, which is a characteristic of *C*. *hyperboreus*. Several hundred females were collected in a 50 L bucket to be distributed into incubation buckets.

### Preparation of incubation water

For the initiation of incubations six 50 L buckets were filled with 0.3 μm filtered sea water obtained from a seawater intake at 80 m depth (*fsw*). For the low pH treatment, small volumes of *fsw* acidified to ca. pH_NBS_ 5.5 (National Bureau of Standards scale) by CO_2_ bubbling (Mapcon CO_2_, Yara Praxair, Tromsø, Norway) were mixed into three of these buckets to reach a target pH_NBS_ of 7.5 as measured by a Metrohm 826 pH meter equipped with a Metrohm LL aquatrode electrode. Total scale pH (pH_T_) was determined spectrophotometrically in 12 mL water samples pipetted in 5 cm quartz cuvettes. Absorbance was measured at 578 nm, 434 nm, and 730 nm using a UV-2401PC spectrophotometer (UV-2401PC, Shimadzu Inc., Kyoto, Japan) after addition of 10 μL of m-cresol-purple. pH_T_ was then calculated according to Clayton and Burne [43] with a dye-addition correction [44] and adjusted from measurement temperature to *in situ* temperature according to Gieskes [45]. We did not measure a second carbonate chemistry variable as in our previous OA studies [17, 32, 33, 46–49], and we were unable to calculate the full carbonate chemistry, which is otherwise recommended [50].

For food, paste of the diatom *Thalassiosira weissflogii* (Tw 1200, Reed Mariculture, Campbell, CA, USA) was added to a final concentration of ca. 10 μg Chl *a* L^-1^. Prior to the experiment, the necessary dilution of the algal paste was established from the Chl *a* content of a 100x dilution of the algal paste determined using a spectrophotometer (UV-2401PC, Shimadzu Inc.) after overnight extraction in 70% ethanol [51]. Cell concentrations in this dilution was established by cell counts under the microscope in a Bürker-Türk counting chamber. The suitability of the algal paste as prey for *C*. *glacialis* had been assured previously by comparing faecal pellets counts from incubations of copepods with counts from copepods incubated at similar concentrations of live *T*. *weissflogii* (Peter Thor, *pers*. *comm*.).

### Copepod and egg incubations

Collected *C*. *glacialis* females were released into 30 L of *fsw*, which was divided in six by volume. Copepods from these six batches were then caught on a 200 μm filter and re-introduced immediately into the six incubation buckets. This started the 7 d incubation of the females. No additional females were added during those 7 days. The water was kept in motion by gentle bubbling and turned once every day to keep algal food in suspension. Ingestion was ascertained by frequent observations of the copepods’ gut colour.

**Fig 1:**
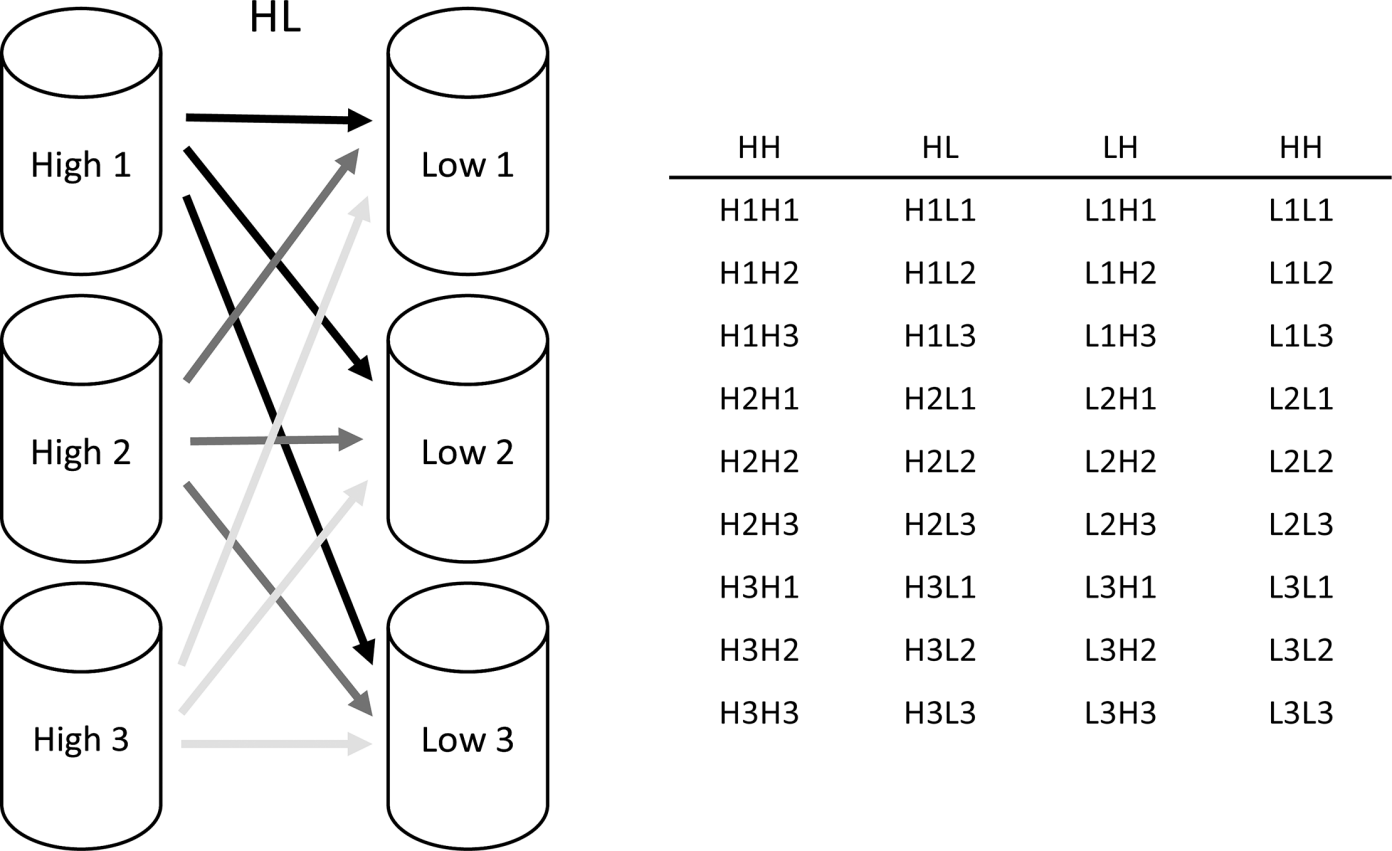
Transplants of eggs among incubation buckets. For clarity only transplants in one treatment group (High to low pH; HL) are shown. The table shows all transplants for each treatment group. Three flasks with eggs were transplanted along each of the arrows in the figure. In total, each treatment group contained nine transplants.

After incubating for 7 d, all eggs were removed from the bottom of the buckets by siphoning and the next day, all eggs produced from day 7 to day 8 were collected from each bucket, also by siphoning. These eggs were collected on a 50 μm mesh size sieve, divided in two and re-suspended in pre-prepared high and low pH incubation water in two 500 mL pitchers. From each of these pitchers, eggs were distributed by volume into nine replicate 50 mL flat cell culture flasks (VWR Collection, Darmstadt, Germany) equipped with 50 μm mesh in the caps to allow exchange with the outside water (total of 2 pH levels x 9 replicates x 6 buckets = 108 flasks). These flasks were then transplanted from the origin bucket to all six incubation buckets according to the design in Fig 1 for the remainder of the experiment. The flask received on average 21 ± 9 eggs (mean ± SD). Eggs were counted twice every day directly in the flasks under a stereomicroscope until the fourth day after which they were counted once every day. Eggs where clearly visible through the flask walls.

pH_T_ was monitored as above once every day in all buckets, and when necessary, the water was renewed by inserting a large 200 μm sieve into the water and siphoning off approximately 3/4 of the water in the bucket from inside of this sieve and replacing it with newly prepared water. Algal concentration were measured by cell counts once every day and algae were added to maintain target concentrations.

### Data treatment and statistical analyses

For each flask, number of remaining eggs was converted to fraction hatched at every sampling event. Egg hatching success (*EHS*) was calculated as the mean of fraction hatched from the last three sampling events. To investigate possible delay effects of pH_T_ in hatching, a sigmoid Hill function was fitted to the cumulative increase in fraction hatched eggs over time in each flask. The Hill function outputs values of average time to hatching (*K*_*m*_) and maximum egg hatching (*E*): $\hat{e}=\frac{Et^{h}}{{K_{m}}^{h}+t^{h}}$, where *ê* is predicted fraction hatched at time *t* and *h* is the sigmoid inflexion factor. After removal of outliers (values further than two standard deviations from the median), values of *EHS* and *K*_*m*_ (interpreted as average time to hatching) were compared among the four treatment groups (high to high pH, high to low pH, low to high pH, and low to low pH) by 1-factor permutational analysis of variance (PERMANOVA) on similarity matrices assembled using Euclidian distances in Primer 6+ [52] employing a nested PERMANOVA design: Treatment group + transplants(treatment group), where “transplants” were the specific transplants among the treatment groups (i.e. the arrows in Fig 1).

Average water temperature and pH_T_ throughout the incubation period were compared among incubation buckets by 1-factor PERMANOVA on similarity matrices assembled using Euclidian distances.

All PERMANOVA tests were followed by PERMDISP tests to verify the assumption of homogeneity of multivariate dispersions.

## Results

There were no significant differences in average temperature among the incubation buckets during the incubation period (Table 1; 1-factor PERMANOVA: Pseudo-F_5,71_ = 0.18, *P* = 0.98). pH_T_ differed significantly between high and low pH treatments and there were no significant difference among buckets within treatments (1-factor PERMANOVA pair-wise tests: *P* > 0.05). There were no significant differences in diatom food concentration among buckets (1-factor PERMANOVA: Pseud-F_5,59_ = 0.29, P = 0.91).

**Table 1:** Averages of temperature (T), total scale pH (pH_T_), salinity (S), and algal concentration during the incubation period.

Bucket	T	pH_T_	S	Algal concentration	
	°C			μgChl *a* L^-1^	cells mL^-1^
High pH 1	5.48±0.22	8.01±0.05	35.12±0.07	10.00±2.92	8100±2371
High pH 2	5.45±0.31	7.96±0.09	35.12±0.04	9.48±1.72	7675±1402
High pH 3	5.51±0.30	7.97±0.05	35.12±0.04	8.74±4.54	7077±3688
Low pH 1	5.58±0.26	7.50±0.13	35.15±0.05	10.14±3.18	8222±2582
Low pH 2	5.52±0.32	7.48±0.15	35.16±0.05	10.10±3.28	8188±2663
Low pH 3	5.54±0.27	7.45±0.18	35.15±0.07	10.08±3.08	8162±2507

Means ± standard deviations.

**Fig 2:**
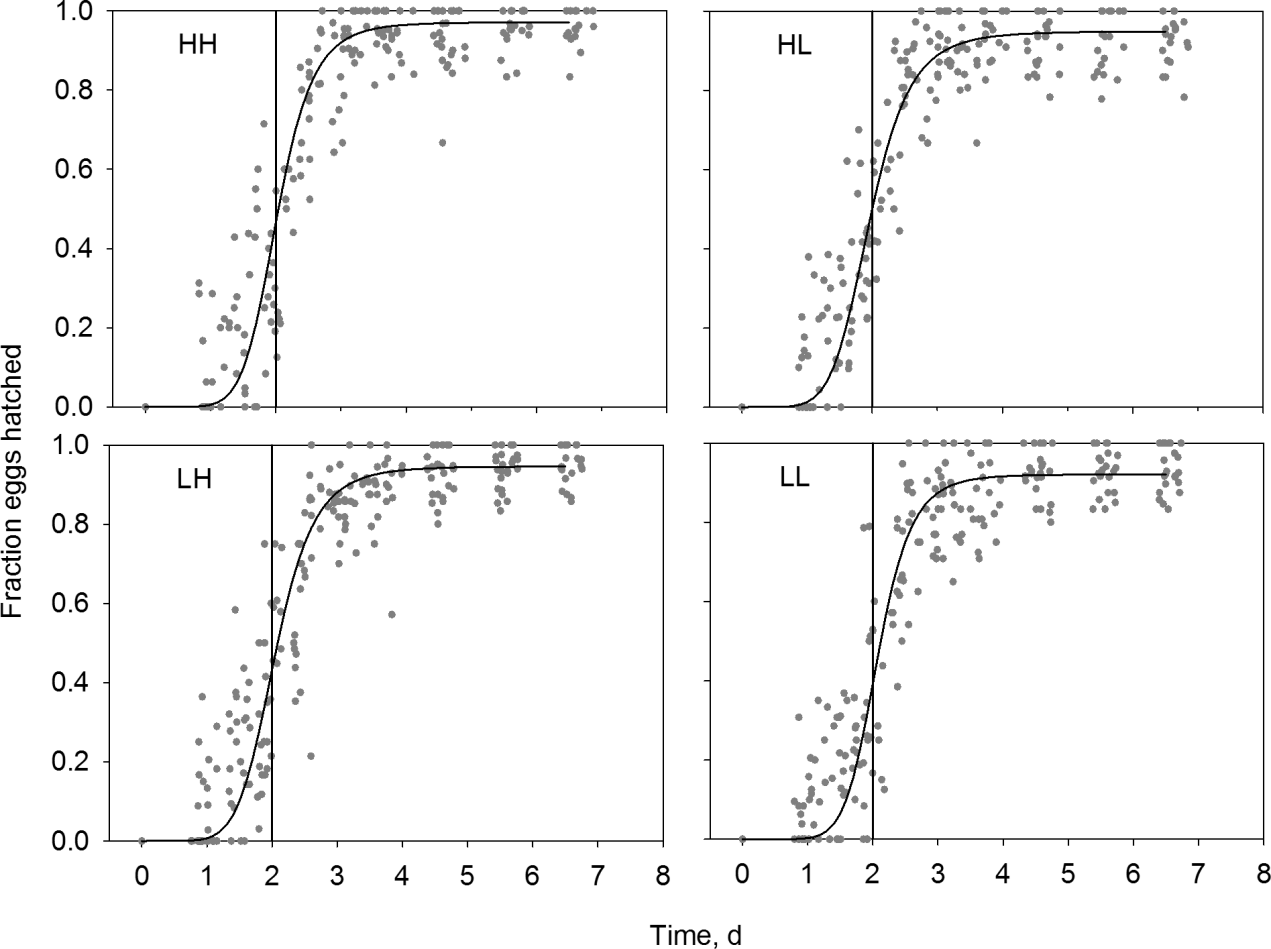
Cumulative egg hatching during the incubation period. To enable comparison among flasks holding different initial numbers of eggs, egg counts were calculated as fractions of the number of eggs at the first count. Solid lines show mean predicted values from the Hill regressions on each individual flask. The vertical lines at t = 2 d is inserted as a guide to facilitate visual judgement of differences among treatments.

In general egg hatching took place two days after collection of eggs from the bucket floors (Fig 2). There were no significant differences in average time to hatching (*K*_*m*_) nor was there any significant confounding effects from the nested transplant factor (1-factor PERMANOVA: Treatment group: Pseudo-F_3,93_ = 0.70, *P* = 0.55, transplant(treatment group) Pseudo-F_32,93_ = 1.61, *P* = 0.057) (Table 2). Furthermore, there were no significant difference among the four treatment groups in egg hatching success (*EHS*) (1-factor PERMANOVA: Treatment group: Pseudo-F_3,93_ = 0.44, *P* = 0.72, transplant (treatment group) Pseudo-F_32,93_ = 0.99, P = 0.51) (Table 2).

**Table 2:** Egg hatching success (*EHS*) and average time to hatching (*K*_*m*_) in the four treatments. Means ± standard deviations. Values of *K*_*m*_ are means of individual regressions on cumulative hatching from each incubation flask.

Treatment	*EHS*	*K*_*m*_
		d
HH	0.94 ± 0.06	2.02 ± 0.23
HL	0.92 ± 0.07	1.97 ± 0.16
LH	0.94 ± 0.05	2.05 ± 0.29
LL	0.89 ± 0.20	2.08 ± 0.24

All raw data on egg hatching are submitted in the supporting S1 Table.

## Discussion

In the study presented here, we did not detect any significant direct or maternally transferred effects of pH levels predicted for the Arctic Ocean in year 2100 on egg hatching success (*EHS*) or egg hatching timing (*K*_*m*_) of *Calanus glacialis* eggs.

Effects of pH on *EHS* has been studied previously in different *Calanus* species. In *C*. *glacialis* egg hatching success was lower at pH_NBS_ 6.9 [36], whereas pH levels more closely mimicking Arctic Ocean predictions for the year 2100 (pH_NBS_ 7.6) left *EHS* unchanged, although high variation within treatments may have masked effects [36]. When subjecting *C*. *finmarchicus* females and eggs to pH_NBS_ 6.95, *EHS* was strongly reduced [53], but in the congener *C*. *helgolandicus EHS* did not change when subjecting the copepods to pH_NBS_ 7.75 [54]. Considering other calanoid genera, the picture looks somewhat different. *EHS* was significantly lowered in *Acartia clausi* at pH_T_ 7.83 compared to pH_T_ 8.03 [55]. Similarly, in *Acartia tonsa EHS* was significantly lowered already at pH_NBS_ 7.81 [56]. However, the copepods for the *A*. *tonsa* study were obtained from commercial cultures in which the carbonate chemistry may be different than in the wild. It may well be that copepods from such cultures could be adapted to an environment with only minor chemical variations and therefore would respond stronger to pH changes. In *Acartia steueri*, *EHS* was significantly depressed at pH 7.3 (no pH scale indicated) when comparing effects through three generations [57]. However, when trying to extract specific information on the effects of long term multigenerational exposure the significant difference disappeared, although this may have been due to low statistical power. A study of OA effects on complete pelagic communities from the Baltic Sea revealed significant negative effects of low pH (pH 7.45) on *EHS* [58, 59]. Interestingly, by transplanting eggs between low and high pH environments, Vehmaa and colleagues showed that maternal effects can alleviate pH effects on *EHS* but only at moderately reduced pH [58]. Eggs from females exposed to moderately reduced pH experienced significantly higher *EHS* compared to eggs produced by non-exposed females, contrary to what we observed in *C*. *glacialis*. Also, decreased pH may act to change the egg hatching response to increasing water temperature. In *Acartia sp*., low pH reduced the positive effect of increased temperature on *EHS* [60]. On the other hand, this interacting effect was not seen in *A*. *clausi* where low pH reduced *EHS* at both high and low temperature [55]. There is no calcification in the copepod exoskeleton and OA effects are carried to copepods foremost by changes in hemolymph pH. Consequently, and because of logistical constraints, we did not measure a second carbonate chemistry variable to enable calculation of the full chemistry. Nevertheless, alkalinity is mostly stable and intermediate in the Kongsfjord [61] and we expect no extremes of carbonate chemistry at the time of the experiments.

We studied egg hatching in *C*. *glacialis* despite the previous findings that *EHS* are not influenced by pH levels similar to the present study [36] because *EHS* is not the only determinant of population recruitment success. Copepods experience widely different predation risk through their life cycle. Eggs and young nauplii larvae constitute important prey for pelagic predators and consequently they experience high mortality rates [37–39]. Older nauplii and copepodite stages possess more efficient behavioural escape mechanisms which lessen size dependent predation risk [62, 63]. Thus, while decreased *EHS* would have direct consequences for population recruitment, any temporal delay in the timing of egg hatching or development in the young stages is equally detrimental since it increases individual life-time predation risk. Moreover, and perhaps more importantly, delayed hatching and development may increase the risk of phenological miss-match with the succession of the phytoplankton bloom [25]. Although *C*. *glacialis* shows capital breeding in the ice-free Kongsfjord, they rely on the relatively short ice algal and later pelagic phytoplankton blooms for the production of larvae in most of the Arctic [41]. While increasing temperatures may at least partially remove the ice algal bloom, they also shifts the pelagic bloom to occur earlier in the season. Copepods experience the same shift, but it not as pronounced resulting in a temporal trophic miss-match, which impairs optimal exploitation of the bloom [25]. Obviously, any delay in egg hatching by OA would only accentuate this miss-match. Hatching rate has been shown to be significantly impaired already at pH_T_ 7.75 in *Pseudocalanus acuspes* [49]. Also, studies on *Acartia sp*. have shown significant delay of egg hatching at low pH [60]. However, a closer look at the Vehmaa et al. data [60] reveals that samples were not taken simultaneously around *K*_*m*_ for the different treatments. This non-simultaneous sampling may have biased results and there could be a risk that differences in the sigmoid accumulation of hatched eggs among treatments emerged as an artefact.

Effects of OA levels predicted for year 2100 on egg hatching seem to be lower in larger than smaller calanoid copepods. Such difference could arise from differences in the sensitivity of energy allocation to OA between the two groups. For instance, energetic expenses of growth seems to increase at decreasing pH in female *P*. *acuspes* [48], and a recent study indicated that this may also be true for *C*. *glacialis* stage IV copepodites [46]. On the other hand, a lack of similar effects in stage V copepodites in that same study showed that effects may be stage dependant and adult females may respond differently [46]. Alternatively, the differences may stem from differences in ontogeny. Copepods of the large *Calanus* genus undergo ontogenetic vertical migration and winter hibernation in the deep. During such overwintering, the copepods experience haemolymph pH possibly as low as 5.5 as a result of metabolic depression during hibernation [64]. *C*. *glacialis* is an opportunistic capital breeder and production of the first eggs of the season often takes place in the deep [41]. It is therefore quite conceivable that mechanisms to counter low pH could have evolved to protect early oogenesis. Later in the season, eggs are predominately produced on ingested energy (income breeding) [40, 41]. Broader support for the idea that taxa living under different pH regimes have evolved different physiologies has been found also in pteropods and polychaetes [65–68].

Naupliar growth and development is similarly unaffected and it seems that *C*. *glacialis* will develop from oviposition to the first copepodite stage unaffected under the future OA scenario tested here [33]. Consequently, OA will not alter the exposure to early life predation mortality or introduce any phenological miss-match with phytoplankton bloom timing. Similarly, larval growth and development of the congener *C*. *finmarchicus* is equally unaffected which adds strength to the argument that naupliar recruitment and development in the *Calanus* genus is generally unaffected by OA [69].

## Supporting information

S1 Table Raw values of unhatched eggs remaining versus time. The first sheet, “info”, contains information on the ordering of the buckets and replicate flasks in the data sheets. Data sheets are arranged according to target buckets in the transplant (H meaning high pH and L meaning low pH). In each sheet the first column shows origin bucket label, the second column shows target bucket label, and the following columns show time (d) and number of unhatched eggs remaining at each sampling event.
(XLSX)
